# (Sr, Ca)AlSiN_3_:Eu^2+^ Phosphor-Doped YAG:Ce^3+^ Transparent Ceramics as Novel Green-Light-Emitting Materials for White LEDs

**DOI:** 10.3390/ma16020730

**Published:** 2023-01-11

**Authors:** Qing Yao, Xinyi Pan, Junjie Tian, Zhihang Chen, Hongbin Ji, Yun Wang

**Affiliations:** 1 School of Mechanical Engineering, Nantong University, Nantong 226019, China; 2 Nantong Cotton Machinery, Nantong 226300, China; 3 School of Mechanical Engineering, Jiangsu University, Zhenjiang 212013, China

**Keywords:** YAG:Ce^3+^ phosphor transparent ceramics, (Sr, Ca)AlSiN_3_:Eu^2+^ phosphors, green-light ceramic, spectral regulation, white-light-emitting diode

## Abstract

In this work, based on Y_3_Al_5_O_12_:Ce^3+^ (YAG:Ce^3+^) transparent ceramic and (Sr, Ca)AlSiN_3_:Eu^2+^ phosphors, novel green-light-emitting materials were systematically studied. YAG:Ce^3+^ transparent ceramics with different doping-concentrations, from 0% to 1% (Sr, Ca)AlSiN_3_:Eu^2+^ phosphors, were fabricated by dry pressing and vacuum sintering. The serial phosphor ceramics had 533 nm green-light emission when excited by 460 nm blue light. The PL, PLE, and chromaticity performances were measured, indicating that more of the green-light component was emitted with the increase in doping concentration. The addition of (Sr, Ca)AlSiN_3_:Eu^2+^ phosphor increased the green-light wavelength area and improved the quantum yield (QY) of the YAG:Ce^3+^ ceramic matrix. The phase composition, microstructure, crystal-field structure and phosphor distribution of (Sr, Ca)AlSiN_3_:Eu^2+^ phosphor-doped YAG:Ce^3+^ transparent ceramics were investigated, to explore the microscopic causes of the spectral changes. Impressively, (Sr, Ca)AlSiN_3_:Eu^2+^ phosphors were distributed homogeneously, and the pinning effect of phosphor caused the suppression of grain growth. The novel materials could provide an effective strategy for full-spectrum white lighting and displaying applications in the future.

## 1. Introduction

YAG:Ce^3+^ phosphor transparent ceramics (TCs) can be excited by approximately 450–460 nm of blue light, to produce white LEDs/LDs [1,2,3]. These ceramics have high lumen-efficiency and thermal stability compared to phosphors in glass (PiG) [4,5,6] and single crystals (SC) [7,8,9]. However, the lack of red- and green-light components causes a low color-rendering-index (CRI) in the spectrum [2,10]. Therefore, to achieve the full spectrum of high-quality white light, the development of green-light and red-light phosphor transparent ceramics is very urgent. Numerous studies have focused on red-light ceramics [11,12,13]. The multicolor-tunable Eu^3+^/Bi^3+^:Y_2_Zr_2_O_7_ transparent ceramic was successfully fabricated using vacuum-sintering technology and strong red (λ_ex_ = 250 nm) and orange (λ_ex_ = 365 nm) emissions, which were also observed at room temperature upon changing the excitation wavelengths [14]. Novel red-emitting ZrO_2_-doped (Gd,Lu)_2_O_3_:Eu transparent phosphor ceramics with a high color-rendering-index close to 90 were fabricated by Li et al. [15]. Additionally, a series of YAG:Ce,Mn transparent ceramics were prepared via a solid-state reaction, and the Mn^2+^–Si^4+^ pairs effectively modulated the emission spectrum by compensating a broad orange-red and red spectrum-component to yield high-quality warm white light [16]. Zhou et al. adopted a Cr/Ce-doped YAG-transparent-ceramic strategy to complement the red spectral component and improve the color-rendering performance [17]. Hence, co-doping red-emitting ions, such as Eu^3+^, Gd^3+^, Mn^3+^, or Cr^3+^, into YAG:Ce^3+^ phosphor transparent ceramics has been considered as an effective way to compensate for the loss of the red component.

However, green-light phosphor transparent ceramics also have an important role and potential in lighting, displaying, and medical treatment [18,19,20]. In the past, we studied green-light ceramics, which were mainly based on Lu_3_Al_5_O_12_:Ce^3+^ (LuAG:Ce^3+^) phosphors ceramics, as the matrix to solve the spectral regulation [21,22,23]. LuAG:Ce^3+^ ceramic phosphors were also regarded as the most promising green color-converter, and the luminous efficacy was promoted to 216.9 lm W^−1^ by designing the Ba^2+^–Si^4+^ pair and air annealing [21]. Wei et al. reported an Al_2_O_3_-LuAG:Ce composite ceramic phosphor for high-brightness laser phosphor display [24]. Likewise, the blue-green emitting Sc^3+^-doped LuAG:Ce phosphor ceramics, as the phosphor converter of high-power LEDs, were successfully prepared using vacuum solid-state sintering, based on the engineering of ion substitution [25]. Nevertheless, green-light phosphor transparent ceramics based on the YAG:Ce^3+^ matrix have rarely been reported. Green-light ceramics with YAG:Ce^3+^ matrix are conducive to co-firing and doping. This will greatly facilitate the preparation of multi-doping and composite structural ceramics, and benefit the regulation of spectra.

In this work, YAG:Ce phosphor transparent ceramics with different doping concentrations of (Sr, Ca)AlSiN_3_:Eu^2+^ phosphors have been fabricated using vacuum sintering, emitting a 533 nm green-light when excited by 460 nm blue-light. Optical properties such as photoluminescence (PL), photoluminescence excitation (PLE), quantum yield (QY), and the chromaticity performance were measured, and the influence of the (Sr, Ca)AlSiN_3_:Eu^2+^ phosphor-doping concentration was discussed systematically. In particular, the phase composition, microstructure, crystal-field structure, and (Sr, Ca)AlSiN3:Eu^2+^ phosphor distribution were investigated, to explore the microscopic causes of the spectral changes. Therefore, the composite transparent ceramics have been designed as novel green-light-emitting materials for white LEDs.

## 2. Experimental Procedure

### 2.1. YAG:Ce-Phosphors Composite Ceramics

In the present research, high-purity commercial α-alumina (Al_2_O_3_, 99.99% purity, D = 160 nm), yttrium oxide (Y_2_O_3_, 99.99% purity, D = 600 nm) and ceria oxide powders (CeO_2_, 99.9% purity, D = 50 nm, all chemicals from Alfa Aesar, Ward Hill, America) were selected as starting materials. They were weighed precisely, in accordance with the (Ce_0_._001_Y_0_._999_)_3_Al_5_O_12_ formula. The commercial (Sr, Ca)AlSiN_3_:Eu^2+^ phosphors (99.9% purity, D = 15 μm, Beijing Yuelong Chemical, Beijing, China) and Y_2_O_3_-Al_2_O_3_-CeO_2_ mixed powders were mixed precisely, using the weight formula *x*(Sr, Ca)AlSiN_3_:Eu^2+^: (*1-x*) (Ce_0_._001_Y_0_._999_)_3_Al_5_O_12_ (*x = 0%, 0.05%, 0.1%, 0.5%, 1%*), denoted as 0SCASNE (only YAG:Ce as a reference), 0.05SCASNE, 0.1SCASNE, 0.5SCASNE and 1SCASNE, respectively. A measure of 0.5 wt.% tetraethyl orthosilicate (TEOS, 99.99%, Alfa Aesar, Ward Hill, MA, America) was chosen as the sintering additive.

**Figure 1:**
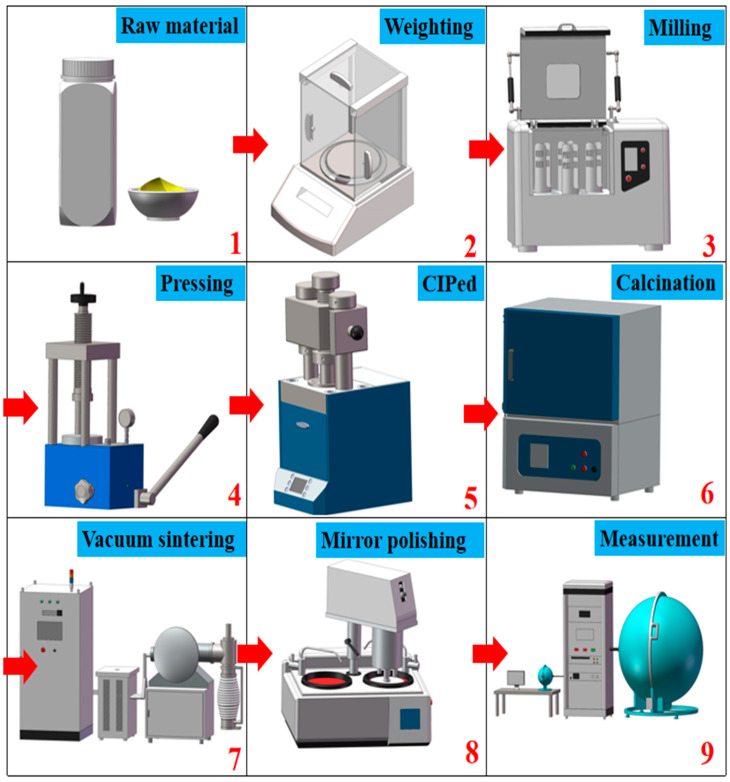
Flowchart for the preparation process of SCASNE phosphor transparent ceramics. The sequence of numbers represents the process of sample preparation and characterization. It has already indicated it on the diagram.

These powders were mixed with absolute ethyl alcohol in a ball-milling jar and then planetary milled for 12 h. The mixture was dried at 60 °C in an oven for 18 h and then meshed and sieved through a 200-mesh screen. After that, the sieved powder mixture was initially uniaxially pressed at 20 MPa in a stainless-steel mold with a diameter of 25 mm, and then cold isostatic pressed (CIPed) at 200 MPa for 300 s, to obtain further compacted powder pellets. The pressed pellets were then calcined at 800 °C for 4 h in air, to remove the organic residues. The calcined green bodies were then sintered in a tungsten-mesh heated vacuum furnace at 1780 °C, for 8 h. After that, the specimens were mirror polished on both surfaces to a thickness of 1.0 mm. The flowchart using the nine-square lattice of the whole preparation process is shown in Figure 1.

### 2.2. Characterization

The crystalline-phase compositions of all samples were characterized using an X-ray diffraction system (XRD, Model D5005, Siemens, Munich, Germany) with a scanning range of 10–80 °C (2θ) and a dwelling time of 0.02 s per step. The crystal-structure refinement was performed using the Rietveld method with the software FULLPROF^TM^. The fracture-surface microstructures and elemental distributions of the ceramics were observed using a scanning electron microscope (SEM, Hitachi, TM-3030 plus) with an energy-dispersive-spectrometer (EDS) system. A confocal laser scanning microscope (CLSM, TCS SP5, Leica, Germany) was used to observe the distribution of phosphors in YAG:Ce^3+^ ceramic matrix. The transmission spectra of the 1 mm-thickness polished specimens were tested using a UV–VIS–NIR spectrophotometer (UV 3600 Plus, Shimadzu, Japan). The QY, PL, and PLE were measured using a fluorescence spectrophotometer (FLS1000, Edinburgh Instruments, Edinburgh, Scotland). An integrating sphere (HPCS6500, HOPOOCOLOR, Hangzhou, China) was used to obtain the chromaticity performance of a series of samples excited by a 460 nm blue chip with 350 mA current and 10 W electric power.

## 3. Results and Discussion

**Figure 2:**
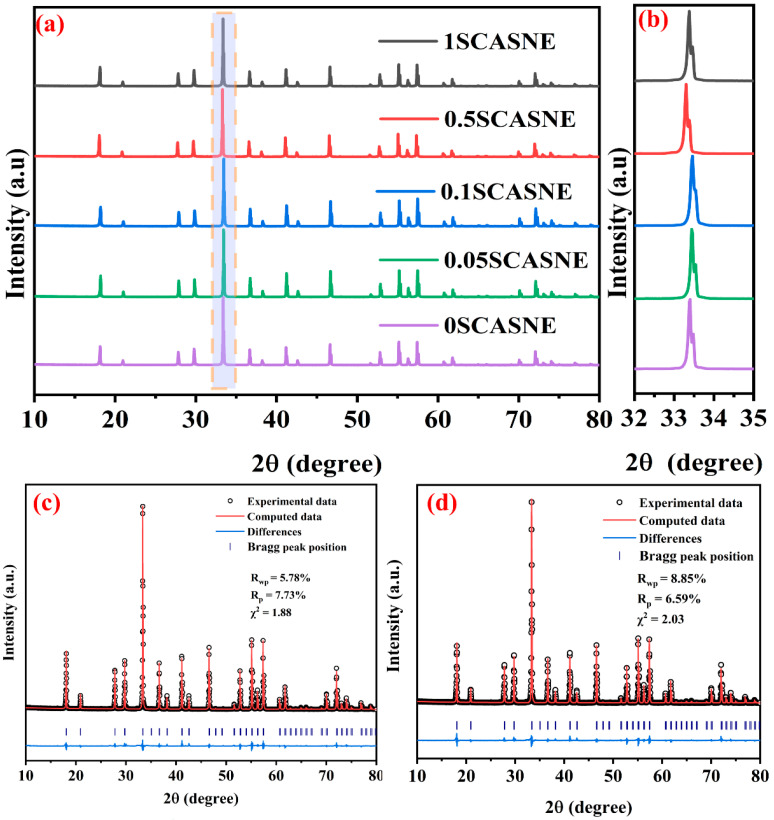
XRD patterns of (**a**) serial SCASNE ceramics with different phosphor-doping concentrations; (**b**) magnified XRD patterns around 33.5°; Rietveld-refinement patterns of (**c**) 0SCASNE and (**d**) 1SCASNE.

Figure 2 shows the XRD patterns and refinement results of the serial SCASNE ceramics. Figure 2a exhibits the XRD patterns of samples from 0SCASNE to 1SCASNE sintered at 1780 °C for 8 h. All the diffraction peaks of the SCASNE ceramics were matched well with the cubic YAG phase (PDF #97-006-7103) [26], and there was no impurity or redundant peaks observed at any (Sr, Ca)AlSiN_3_:Eu^2+^ phosphor-doping concentration (from 0% to 1%). This indicated that the phosphors were a complete solid solution in the host YAG lattice. As could be seen from the magnified diffraction peaks around 33.5 °C (shown in Figure 2b), all the diffraction peaks of the SCASNE ceramics were shifted to lower angles. This demonstrated that (Sr, Ca)AlSiN_3_:Eu^2+^ phosphor doping resulted in a larger unit cell [27,28]. To further acquire the effect of phosphor doping on the YAG:Ce^3+^ crystal structure, crystal-structure refinement was performed using the Rietveld method with the software FULLPROF^TM^ [29]. The refinement results of 0SCASNE and 1SCASNE are presented in Figure 2c,d, respectively. Because χ^2^ = 1.88 and χ^2^ = 2.03 < 10, this indicated that refinement was effective. The 0SCASNE lattice constant was a = b = c = 12.0074 Å, α = β = γ = 90 °C while the 1SCASNE lattice constant was a = b = c = 12.0086 Å, α = β = γ = 90 °C. The larger lattice size coincided with the trend in Figure 2b, owing to the bigger ionic of (Sr, Ca)AlSiN_3_:Eu^2+^ phosphors in the YAG:Ce^3+^ host lattice.

**Figure 3:**
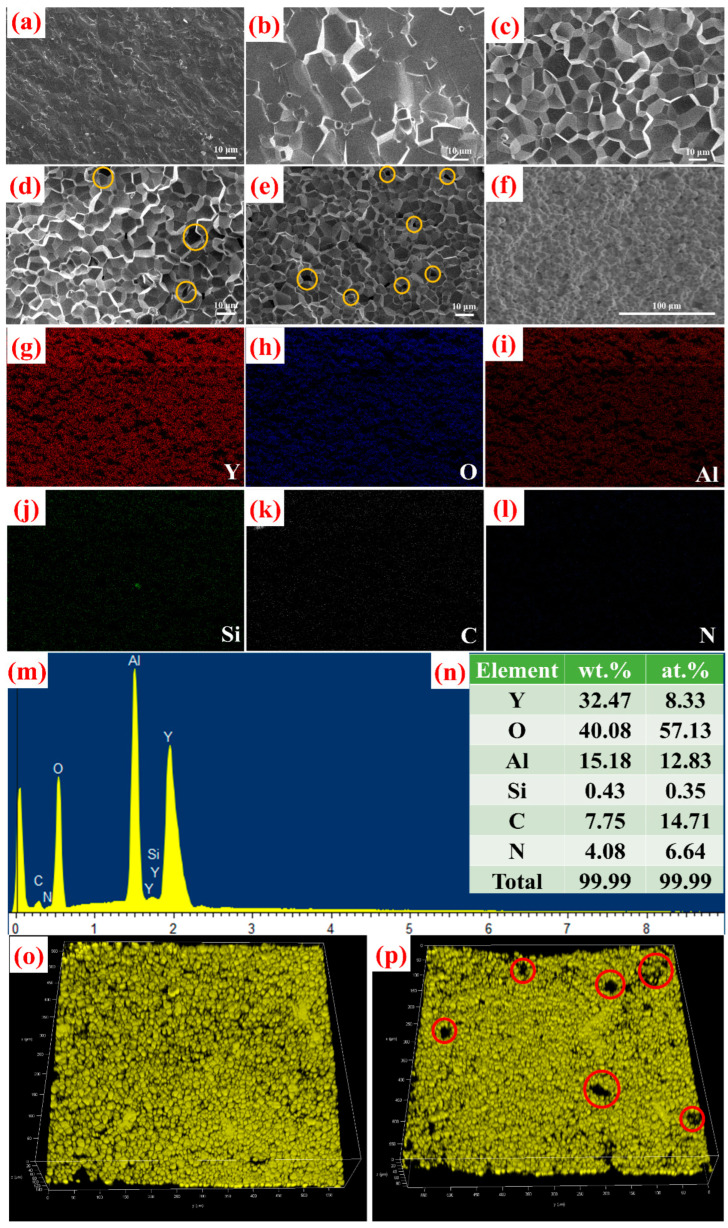
Microscopic information of the prepared samples. SEM images of the fracture surfaces of (**a**) 0SCASNE, (**b**) 0.05SCASNE, (**c**) 0.1SCASNE, (**d**) 0.5SCASNE and (**e**) 1SCASNE. (**f**) SEM image of 1SCASNE for EDS mapping, and (**g**–**n**) EDS elemental-mapping and proportion-analysis images of 1SCASNE; 3D-reconstruction CLSM images of (**o**) 0.05SCASNE and (**p**) 1SCASNE, respectively.

The SEM micrographs of the fracture surfaces of the prepared ceramics 0SCASNE to 1SCASNE are exhibited in Figure 3a–e. The samples displayed regular grains and clean grain boundaries. As the phosphor concentration increased, the fracture surface morphologies changed from transgranular to intergranular. Additionally, the micro-pores could be more easily observed (marked with yellow circles in Figure 3d,e), resulting in a decrease in their transmittance consistent with the optical-transmittance spectra shown in Figure 6b. Moreover, the micro-pores could act as scattering centers in phosphor ceramics, improving the utilization of incident blue-light. The source of micro-pores must be the position of the original phosphor dissolved into the ceramic. In addition, the grain sizes were significantly smaller with the increase in the doping concentration. This indicated that the pinning effect of the phosphor on the grain boundary caused the suppression of grain growth [30,31,32].

To further understand the element distribution within the samples, the EDS analysis of the 1SCASNE phosphor ceramics was recorded, and is shown in detail in Figure 3f–n. The six elements of Y, O, Al, Si, C and N were distributed homogeneously, implying that phosphor (Sr, Ca)AlSiN_3_:Eu^2+^ and the ceramic matrix were fully mixed and made a solid solution. However, the weighing percentage of six elements only reached 99.99%, regardless of mass or atoms. It was most likely that some doping concentrations such as Ce^3+^ and Eu^2+^ were very low, which was beyond the detection limit of the EDS.

In order to explore the existence and distribution of luminescent trace ions in the sample 1SCASNE, the 3D reconstruction CLSMs of 0.05SCASNE and 1SCASNE are further shown in Figure 3o,p, respectively. The phosphors are uniform in Figure 3o, while some micro-pores appear in Figure 3p (marked within red circles). As the phosphor-doping concentration increased, it was supposed that certain phosphor substances had been removed under vacuum-sintering conditions.

**Figure 4:**
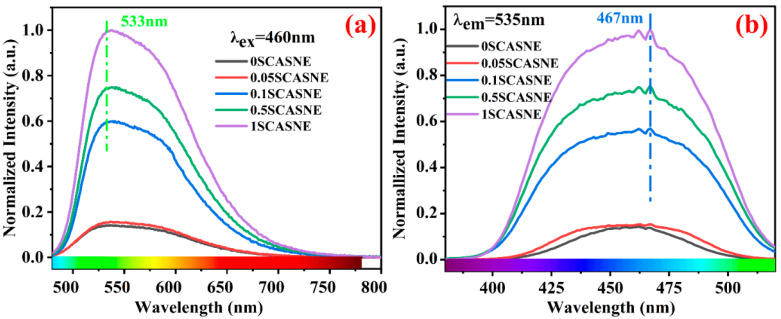
Normalized (**a**) PL and (**b**) PLE spectra of the prepared serial SCASNE phosphor ceramics.

Figure 4 indicates the PL and PLE spectra of the prepared serial-SCASNE phosphor ceramics. From Figure 4a, showing the PL (λ_ex_ = 460 nm) spectra, it was clear that the SCASNE phosphor ceramics had broadband emission centered at 533 nm under 460 nm excitation. This was a “blue-shift”, compared to the conventional typical Ce^3+^:5d→4f emission spectra [33,34,35]. Additionally, as the (Sr, Ca)AlSiN_3_:Eu^2+^ doping concentration increased (from 0SCASNE to 1SCASNE), the emission power became stronger, and the full width at half maximum (FWHM) was broader. The peak of the emission spectrum was 533 nm, which was in the wavelength range of the green-light wave band. Correspondingly, Figure 4b of the PLE spectra shows the excitation-spectrum peaks at 467 nm, mainly originating from the Ce^3+^: 4f→5d transitions under an emission peak of 535 nm. As the (Sr, Ca)AlSiN_3_:Eu^2+^ doping concentration increases, the emission power becomes stronger, and the FWHM increases.

**Figure 5:**
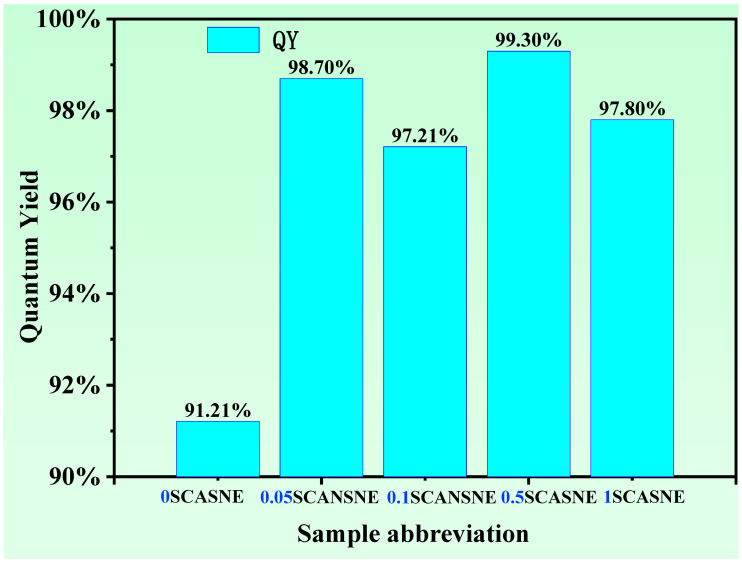
Quantum yield of *x*SCASNE phosphor ceramics (*x* = 0, 0.05, 0.1, 0.5 and 1) and the inset screenshot of the 0.5SCASNE-ceramic test curve.

The quantum yield (QY) values of a series of SCASNE phosphor ceramics were studied and are provided in Figure 5. Under the excitation of 460 nm blue light, the QY values were 91.21%, 98.70%, 97.21%, 99.30%, and 97.80%, respectively, for a 0%, 0.05%, 0.1%, 0.5%, and 1% (Sr, Ca)AlSiN_3_:Eu^2+^ phosphor-doping concentration. It was found that the QY of YAG:Ce^3+^ matrix ceramics were greater than 90%, and (Sr, Ca)AlSiN_3_:Eu^2+^ phosphor enhanced the YQ, due to a higher energy-transfer efficiency [36]. The maximum QY at a 0.5% doping concentration was 99.30%, and the corresponding sample was 0.5SCASNE. This demonstrated that (Sr, Ca)AlSiN_3_:Eu^2+^ phosphor was competent as an efficient dopant for improving the QY of the serial-SCASNE phosphor ceramic.

**Figure 6:**
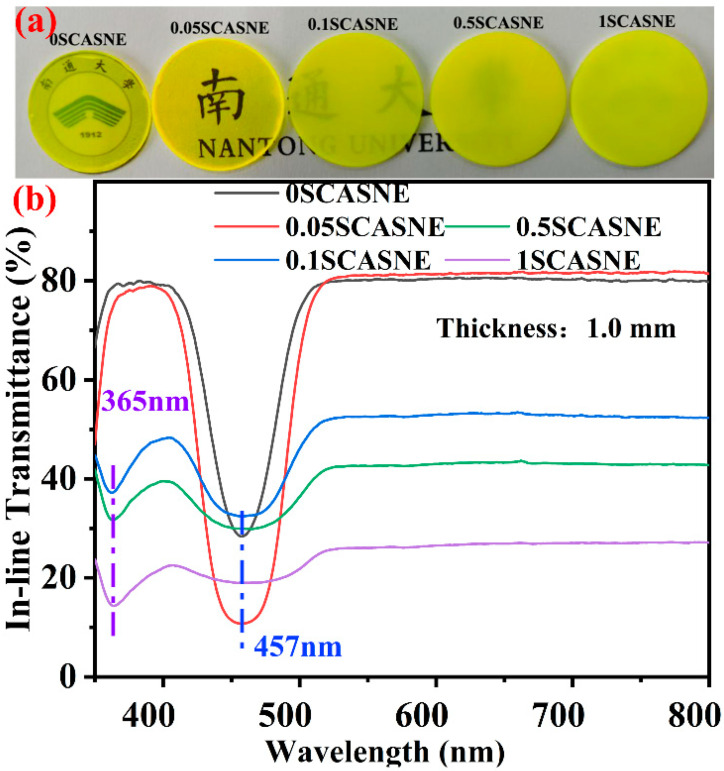
(**a**) Photograph of serial SCASNE ceramics with 1 mm thickness under daylight and (**b**) the optical-transmittance spectra under 350–800 nm wavelengths.

Figure 6 presents the photographs of the serial SCASNE ceramics with a 1 mm thickness and the optical- transmittance spectra under a 350–800 nm wavelength. In Figure 6a, with the increase in (Sr, Ca)AlSiN_3_:Eu^2+^ phosphor, the black logo behind the SCASNE ceramics becomes more and more blurred. It was confirmed that the increase in phosphor concentration promoted the transition from transparent/translucent to basically opaque SCASNE ceramics. This was consistent with the in-line transmittance spectrum presented in Figure 6b. When the contents of the phosphor were 0%, 0.05%, 0.1%, 0.5%, and 1%, the transmittances were 79.71%, 81.35%, 52.35%, 42.74%, and 27.09% @800 nm, respectively. It could be speculated that the phosphor acts as the second phase and scattering centers in the YAG:Ce^3+^ ceramic matrix, to reduce their transmission [37,38]. Meanwhile, the introduction of phosphor also affected the absorption. When the content of doped phosphor was low (0% and 0.05%), the absorption band was observed at 457 nm for 0SCASNE and 0.05SCASNE. This was mainly due to the 4f→5d-level electron transition of the Ce^3+^ ion [39,40,41]. When the phosphor made up more of the content (0.1%, 0.5% and 1%), the absorption band was mainly 365 nm, and the original absorption at 457 nm was not as strong as for the low concentration of doping. This implied that the high concentration of phosphor-doping changed the matrix crystal-field structure [42,43,44].

**Figure 7:**
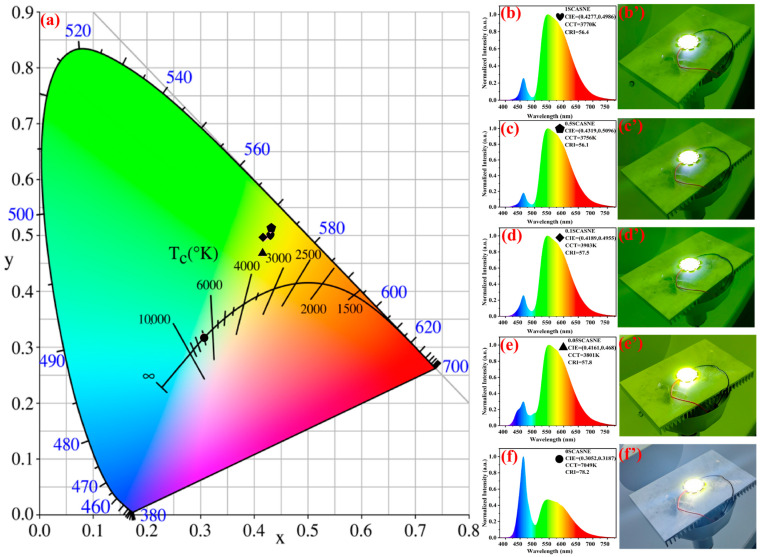
Chromaticity parameters, electroluminescence (EL) spectra, and corresponding photographs of white LEDs driven by a 350 mA current: (**a**) the color coordinates, (**b**/**b’**) 1SCASNE, (**c**/**c’**) 0.5SCASNE, (**d**/**d’**) 0.1SCASNE, (**e**/**e’**) 0.05SCASNE, and (**f**/**f’**) 0SCASNE. Heart, pentagon, diamond, triangle and circle represent the color coordinates of 1SCASNE, 0.5SCASNE, 0.1SCASNE, 0.05SCASNE, 0SCASNE, respectively.

The chromaticity parameters, EL spectra and corresponding photographs of LEDs driven by a 350 mA current and 10 V voltage, are shown in Figure 7. With (Sr, Ca)AlSiN_3_:Eu^2+^ phosphor increased from 0% to 1%, and the CIE color coordinates ranged from (0.3052, 0.3187) to (0.4277, 0.4986) in Figure 7a, corresponding to the pale-white and yellow-green areas, respectively. Obviously, the green light produced by the composite ceramics was consistent with the emission spectrum of Figure 4a. Meanwhile, the CCT of white LED varied from 7049 K to 3750 K, which gradually changed from cool colors to warm colors. The EL and test photographs are provided in Figure 7b/b’–f/f’. Remote-excited white LEDs were constructed by combining the prepared serial-SCASNE ceramics with a blue chip excited at 460 nm, to further evaluate the EL performance. Notably, it was evident that more of the green-light component was emitted from the 0SCASNE ceramics to the 1SCASNE samples, with the obtained CCT of Figure 7a showing the same tendency. The addition of (Sr, Ca)AlSiN_3_:Eu^2+^ could increase the width of 525 nm–625 nm, and the green-light wavelength area increased significantly. Additionally, (Sr, Ca) AlSiN_3_:Eu^2+^ could be used as the second phase, to improve the blue-light utilization. The results showed that green light could be obtained by optimizing the (Sr, Ca)AlSiN_3_:Eu^2+^ phosphor concentration in the YAG:Ce^3+^ ceramic matrix.

## 4. Conclusions

In this paper, 0.1% YAG:Ce^3+^ phosphor transparent ceramics with different doping concentrations from 0% to 1% (Sr, Ca)AlSiN_3_:Eu^2+^ phosphors were fabricated by dry pressing and vacuum sintering. The serial-SCASNEs phosphor ceramics had 533 nm green-light emission when excited by 460 nm blue light. The PL, PLE, and chromaticity performance were measured, indicating that more of the green-light component was emitted from the 0SCASNE to the 1SCASNE ceramic samples. The addition of (Sr, Ca)AlSiN_3_:Eu^2+^ phosphor increased the green-light wavelength area and improve the QY of the YAG:Ce^3+^ ceramic matrix. Moreover, information from SEM, EDS, and CLSM showed that (Sr, Ca)AlSiN_3_:Eu^2+^ phosphors were distributed homogeneously, and were cleaned under vacuum-sintering conditions. Impressively, the pinning effect of phosphor caused the suppression of grain growth. The novel doped-phosphor and crystal-field design provided an interesting perspective for creating the green light.

## Data Availability

All data included in this study are available upon request by contact with the corresponding author.
